# Prepregnancy smoking and the risk of gestational diabetes requiring insulin therapy

**DOI:** 10.1038/s41598-020-70873-7

**Published:** 2020-08-17

**Authors:** Mee Kyoung Kim, Kyungdo Han, Sang Youn You, Hyuk-Sang Kwon, Kun-Ho Yoon, Seung-Hwan Lee

**Affiliations:** 1 Division of Endocrinology and Metabolism, Department of Internal Medicine, Yeouido St. Mary’s Hospital, College of Medicine, The Catholic University of Korea, Seoul, 07345 South Korea; 2 Department of Statistics and Actuarial Science, Soongsil University, Seoul, 06978 South Korea; 3 College of Medicine, The Catholic University of Korea, Seoul, 06591 South Korea; 4 Division of Endocrinology and Metabolism, Department of Internal Medicine, Seoul St. Mary’s Hospital, College of Medicine, The Catholic University of Korea, #222 Banpo-daero, Seocho-gu,, Seoul, 06591 South Korea; 5 Department of Medical Informatics, College of Medicine, The Catholic University of Korea, Seoul, 06591 South Korea

**Keywords:** Gestational diabetes, Epidemiology

## Abstract

The relationship between maternal smoking and gestational diabetes mellitus (GDM) is inconclusive. We investigated whether prepregnancy smoking is a risk factor for insulin-requiring GDM in Korean women. Using the National Health Insurance Service database, 325,297 women who delivered between 2011 and 2015 and who received a health examination within 52 weeks before pregnancy were included. Insulin-requiring GDM was defined as no claims for diabetes mellitus and a fasting blood glucose level of < 126 mg/dL before pregnancy, and initiation of insulin treatment during pregnancy. Smoking status was identified in a self-reported questionnaire completed during the health examination. There were 2,114 women (0.65%) with GDM who required insulin therapy. Compared with nonsmokers, the fully adjusted odd ratios (ORs) of former smokers and current smokers for insulin-requiring GDM were 1.55 (95% confidence interval [CI] 1.27–1.90) and 1.73 (1.42–2.09), respectively. The ORs (95% CIs) of insulin-requiring GDM among women who reported ≤ 2, 2–≤ 4, 4–≤ 6, 6–≤ 8, 8–≤ 10, and > 10 pack-years of smoking were 1.50 (1.22–1.84), 1.71 (1.31–2.22), 1.60 (1.13–2.26), 1.97 (1.14–3.40), 2.34 (1.22–4.51), and 2.29 (1.25–4.22), respectively, compared with nonsmokers (*P* for trend < 0.001). This association was similar in women with or without obesity and abdominal obesity. In conclusions, women who smoke have a significantly higher risk of GDM requiring insulin therapy, which may be proportional to the cumulative exposure to smoking. Cessation of smoking should be emphasized in women of childbearing age for the prevention of GDM.

## Introduction

Gestational diabetes mellitus (GDM) is a common medical condition during pregnancy and represents a leading cause of adverse pregnancy outcomes worldwide. The number of women being diagnosed with GDM has increased in the past decades, and this increase is largely attributable to increased obesity and age of pregnant women^1^. Behavioral risk factors for GDM are not well understood, and the relationship between prepregnancy smoking and GDM is inconclusive^2–5^. Some of the previous population-based cohort studies have found a positive relationship between cigarette smoking during pregnancy and GDM^2,3^, whereas other cohort and cross-sectional studies have not^4,5^.

The smoking rate has increased among adolescent and young women worldwide^6^. During the past 25 years, the prevalence of smoking in Korean men decreased in all age groups but increased from 1.6 to 4.0% in Korean women aged 19–34 years^6^. From the public health perspective, recognizing behavioral risk factors is especially important because these factors may be modifiable through appropriate interventions. Having more precise information about how smoking influences GDM will provide a basis for tailoring lifestyle advice for those at higher risk of developing GDM.

The severity of GDM is associated with maternal glucose level that present a positive and direct correlation with the risk of fetal involvement^7^. The need for insulin therapy might be a starting point for the characterization of patients with more severe GDM related to greater difficulty in achieving glycemic control^7^. Therefore, it is important to identify and mitigate any potentially avoidable risks for insulin-requiring GDM. The present study used a nationwide population-based cohort from the National Health Insurance Service (NHIS) database of Korea to examine the relationship between smoking and insulin-requiring GDM.

## Methods

### Data source and study population

Details of the NHIS cohort design, methods, and validity of the records are available in previous studies^8–10^. Briefly, the NHIS is managed by the government and is the single insurer for health-care services; it has a coverage rate of ~ 97% in the Republic of Korea. The NHIS database is available for population-based cohort studies, and information on demographics, national health screening data, diagnosis statements defined by the International Classification of Disease 10th revision (ICD-10) codes, medical treatment, and drug prescription is routinely collected and undergoes quality control before being released for research purpose. As part of the health screening examination, participants provide blood samples and their lifestyle patterns, past medical and family history via a self‐reported questionnaire, and anthropometric measurements such as body weight, waist circumference (WC), and blood pressure are recorded. Enrollees in the NHIS are recommended to undergo a standardized medical examination at least every 2 years.

**Figure 1:**
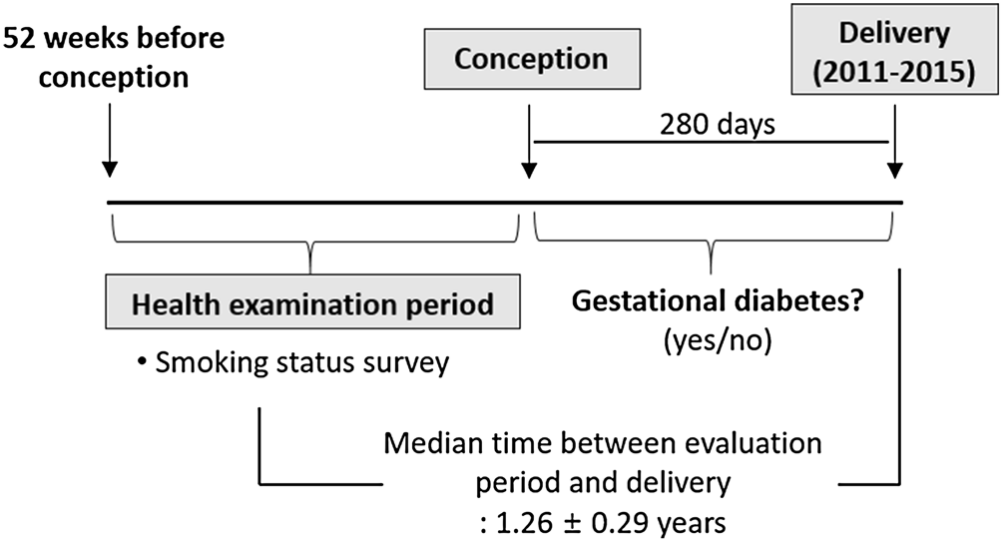
Timeline for the study data collection.

We used the NHIS database to identify women who had delivered between 2011 and 2015. In our study, 280 days before the delivery date was regarded as the date of conception. A total of 329,675 women who had had received a health checkup during the 52 weeks before conception were selected (Fig. 1). Women taking a glucose-lowering agent before pregnancy (n = 2,187), having a fasting glucose level ≥ 126 mg/dL at the health checkup (n = 1,346), or with missing data (n = 845) were excluded. Finally, 325,297 women without diabetes were included in this study. All procedures performed in studies involving human participants were in accordance with the ethical standards of the Helsinki Declaration. This study was approved by the Institutional Review Board (IRB) of Seoul St. Mary’s Hospital, The Catholic University of Korea (No. KC19ZESI0586). Informed consent was waived by IRB because anonymous and deidentified information was used for the analysis.

### Smoking status

At the health checkup, the participants completed a self-reported health survey questionnaire^11,12^. Smoking status was classified as follows: current smoker, defined as those who had smoked > 5 packs (a total of 100 cigarettes) throughout their lifetime and continued to smoke; former smoker, defined as those who had smoked > 5 packs throughout their lifetime but had quit smoking; and nonsmoker, defined as those who had smoked ≤ 5 packs. Both former smokers and current smokers recorded the total duration of smoking (years) and the average number of cigarettes smoked daily. The cumulative lifetime smoking exposure was reported as pack-years by multiplying the average cigarette consumption per day (packs) and the smoking period (years).

### Measurements and definitions

Body mass index (BMI) was calculated by dividing the weight in kilograms by height in meters squared. Obesity was defined as a BMI ≥ 25.0 kg/m^2^ according to the World Health Organization Western Pacific Region guideline^13^. Abdominal obesity was defined as a WC ≥ 85 cm according to the criterion defined by the Korean Society for the Study of Obesity^14^. Information on alcohol consumption (heavy alcohol consumption defined as ≥ 30 g/day) was obtained from the questionnaire. Regular exercise was defined as > 30 min of moderate physical activity performed ≥ 5 times per week or > 20 min of strenuous physical activity performed ≥ 3 times per week. Household income level was dichotomized at the lowest 25%. Blood samples were drawn after an overnight fast, and the serum fasting blood glucose (FBG), lipid, and creatinine levels were measured. People having FBG levels of 100–125 mg/dL were defined as impaired fasting glucose (IFG). Estimated glomerular filtration rate (eGFR) was calculated using the equation from the Modification of Diet in Renal Disease study: eGFR = 175 × serum creatinine^–1.154^ × age^–0.203^ × 0.742. Hospitals that performed these health examinations were certified by the NHIS and were subject to regular quality control.

GDM requiring insulin therapy was defined as having no history of previous diabetes and receiving a prescription of insulin during the pregnancy period. Participants with non-GDM or GDM without insulin treatment were regarded as the control group.

### Statistical analysis

The data are presented as mean ± standard deviation (SD), median (25–75%), or n (%). Student’s *t* test or chi-squared test was used to compare differences in clinical and biochemical characteristics between the insulin-requiring GDM group and control group. Odds ratios (ORs) and 95% confidence intervals (CIs) for GDM were obtained using multiple logistic regression analysis. Multivariable-adjusted analysis was performed to control for the confounding effects of known risk factors for GDM. Model 1 was adjusted for age, alcohol consumption, regular exercise, and income status. Model 2 was adjusted further for baseline BMI, fasting glucose level, family history of diabetes, and dyslipidemia. Potential effect modifications by general obesity, abdominal obesity and baseline glucose tolerance status were identified using stratified analysis and interaction testing using the likelihood-ratio test. Statistical analyses were performed using SAS version 9.4 (SAS Institute Inc., Cary, NC, USA), and a *P* value < 0.05 was considered to indicate significance.

### Prior presentation

This study was presented in abstract form at the Korean Diabetes Association’s 33rd spring scientific congress, Korea, 8–9 May 2020.

## Results

### Clinical characteristics according to the presence of insulin-requiring GDM

There were 2,114 women (0.65%) with GDM who received insulin therapy. These women were older, more obese, had a higher prevalence of hypertension and metabolic syndrome, and were more likely to have a family history of diabetes mellitus (DM) compared with the control group. Women with GDM who received insulin therapy also had higher levels of FBG, total cholesterol, triglycerides, and low-density lipoprotein cholesterol, and lower high-density lipoprotein (HDL) cholesterol level. The percentage of former or current smokers was significantly higher in the insulin-requiring GDM group than in the control group (Table 1).

**Table 1:** Clinical characteristics of the study subjects before pregnancy.

	Control	GDM requiring insulin therapy	*P* value
N	323,183	2,114	
**Age (years)**	29.6 ± 3.6	32.1 ± 4.3	< 0.001
< 35 years	295,315 (91.4)	1,590 (75.2)	< 0.001
≥ 35 years	27,868 (8.6)	524 (24.8)	
**Smoking**			< 0.001
Non	298,331 (92.3)	1795 (84.9)	
Former smoker	12,031 (3.7)	143 (6.8)	
Current smoker	12,821 (4.0)	176 (8.3)	
Heavy alcohol consumption	6,950 (2.2)	47 (2.2)	0.274
Regular exercise	34,029 (10.5)	248 (11.7)	0.075
Income (lower 25%)	64,921 (20.1)	442 (20.9)	0.348
Family history of DM	30,687 (13.2)	477 (29.2)	< 0.001
**BMI (kg/m**^**2**^**)**	20.8 ± 2.7	23.4 ± 4.1	< 0.001
< 18.5	55,441 (17.2)	127 (6.0)	
18.5–22.9	214,553 (66.4)	1,037 (49.1)	
23–24.9	30,163 (9.3)	329 (15.6)	
25–29.9	19,629 (6.1)	465 (22.0)	
≥ 30	3,397 (1.1)	156 (7.4)	
Waist circumferences (cm)	69.3 ± 7.0	75.5 ± 9.7	< 0.001
FBG (mg/dL)	87.3 ± 9.1	95.5 ± 12.1	< 0.001
TC (mg/dL)	177.0 ± 28.8	190.2 ± 34.5	< 0.001
HDL cholesterol (mg/dL)	63.7 ± 14.3	57.9 ± 13.4	< 0.001
LDL cholesterol (mg/dL)	98.5 ± 32.1	111 ± 38.6	< 0.001
Triglyceride (mg/dL)	65 (50–88)	90 (64–132)	< 0.001
eGFR (ml/min/1.73 m^2^)	101.0 ± 33.6	100.7 ± 38.2	0.766
Systolic BP (mmHg)	110.0 ± 10.8	113.7 ± 12.3	< 0.001
Diastolic BP (mmHg)	69.2 ± 8.1	71.8 ± 8.9	< 0.001
Hypertension	4,258 (1.3)	93 (4.4)	< 0.001
Metabolic syndrome	7,865 (2.4)	414 (19.6)	< 0.001

Data are expressed as the mean ± SD, median (25–75%), or n (%).

BMI, body mass index; BP, blood pressure; DM, diabetes mellitus; eGFR, estimated glomerular filtration rate; GDM, gestational diabetes mellitus; FBG, fasting blood glucose; HDL, high-density lipoprotein; LDL, low-density lipoprotein; TC, total cholesterol.

### Risk of insulin-requiring GDM according to the duration and amount of smoking

Using the nonsmokers as the reference group, the multivariable-adjusted ORs (95% CIs) of former and current smokers for insulin-requiring GDM were 1.55 (1.27–1.90) and 1.73 (1.42–2.09), respectively. In both former and current smokers, more amount of smoking was associated with a higher incidence rate and OR of insulin-requiring GDM. Compared with nonsmokers, the ORs of insulin-requiring GDM were higher in former and current smokers in women who smoked ≥ 15 cigarettes/day than in women who smoked < 15 cigarettes/day. The ORs (95% CIs) were 2.42 (1.36–4.31) in former smokers and 2.35 (1.46–3.79) in current smokers who smoked ≥ 15 cigarettes/day and 1.49 (1.21–1.84) in former smokers and 1.65 (1.35–2.03) in current smokers who smoked < 15 cigarettes/day. In current smokers, a long duration of smoking was associated with a higher OR of insulin-requiring GDM; the ORs (95% CIs) were 1.38 (0.82–2.30) and 1.78 (1.45–2.19) for women who smoked cigarettes for < 5 years and ≥ 5 years, respectively (Table 2).

**Table 2:** Adjusted odd ratios and 95% confidence intervals of gestational diabetes mellitus requiring insulin therapy by smoking status, amount and duration.

	n (%)	Events (n)	Incidence rate^a^	Model 1	Model 2
**Smoking status**					
Nonsmoker	300,126 (92.3)	1,795	6.0	1 (ref.)	1 (ref.)
Former smoker	12,174 (3.7)	143	11.7	1.83 (1.54, 2.18)	1.55 (1.27, 1.90)
Current smoker	12,997 (4.0)	176	13.5	2.36 (2.01, 2.77)	1.73 (1.42, 2.09)
**Amount of smoking (no. of cigarettes smoked per day)**					
Nonsmoker	300,126 (92.3)	1,795	6.0	1 (ref.)	1 (ref.)
Former smoker					
< 15	11,454 (3.5)	127	11.1	1.75 (1.46, 2.11)	1.49 (1.21, 1.84)
≥ 15	720 (0.2)	16	22.2	2.93 (1.77, 4.85)	2.42 (1.36, 4.31)
Current smoker					
< 15	11,736 (3.6)	150	12.8	2.26 (1.90, 2.68)	1.65 (1.35, 2.03)
≥ 15	1,261 (0.4)	26	20.6	3.27 (2.19, 4.88)	2.35 (1.46, 3.79)
**Duration of smoking (years)**					
Nonsmoker	300,268 (92.3)	1,797	6.0	1 (ref.)	1 (ref.)
Former smoker					
< 5	4,816 (1.5)	50	10.4	1.93 (1.45, 2.56)	1.60 (1.14, 2.23)
≥ 5	7,265 (2.2)	92	12.7	1.78 (1.43, 2.21)	1.53 (1.20, 1.95)
Current smoker					
< 5	2,347 (0.7)	19	8.1	1.84 (1.16, 2.90)	1.38 (0.82, 2.30)
≥ 5	10,601 (3.3)	156	14.7	2.43 (2.05, 2.89)	1.78 (1.45, 2.19)
**Amount × duration of smoking (pack-years)**					
Nonsmoker	300,126 (92.3)	1,795	6.0	1 (ref.)	1 (ref.)
≤ 2	13,625 (4.2)	139	10.2	1.86 (1.56, 2.21)	1.50 (1.22, 1.84)
2 <, ≤ 4	6,210 (1.9)	81	13.0	2.21 (1.76, 2.77)	1.71 (1.31, 2.22)
4 <, ≤ 6	3,222 (2.0)	49	15.2	2.23 (1.67, 2.98)	1.60 (1.13, 2.26)
6 <, ≤ 8	1,085 (0.3)	18	16.6	2.20 (1.37, 3.53)	1.97 (1.14, 3.40)
8 <, ≤ 10	568 (0.2)	16	28.2	2.97 (1.76, 5.00)	2.34 (1.22, 4.51)
> 10	461 (0.1)	16	34.7	3.27 (1.96, 5.45)	2.29 (1.25, 4.22)

Model 1: Adjusted for age, alcohol drinking, regular exercise, and income status.

Model 2: Adjusted for model 1 + body mass index, fasting blood glucose, family history of diabetes, and dyslipidemia.

^a^Per 1,000 person-years.

Analysis of both the amount and duration of smoking showed a dose–response relationship between pack-years of smoking and the incidence rate or the risk of insulin-requiring GDM. Even ≤ 2 pack-years of smoking was significantly associated with an increased risk of insulin-requiring GDM. In the multivariable-adjusted model, the ORs (95% CIs) of insulin-requiring GDM among women who smoked ≤ 2, 2–≤ 4, 4–≤ 6, 6–≤ 8, 8–≤ 10, and > 10 pack-years were 1.50 (1.22–1.84), 1.71 (1.31–2.22), 1.60 (1.13–2.26), 1.97 (1.14–3.40), 2.34(1.22–4.51), and 2.29 (1.25–4.22), respectively, compared with nonsmokers (*P* for trend < 0.001) (Table 2).

### Effect of smoking on the risk of insulin-requiring GDM according to obesity and glucose tolerance status

**Figure 2:**
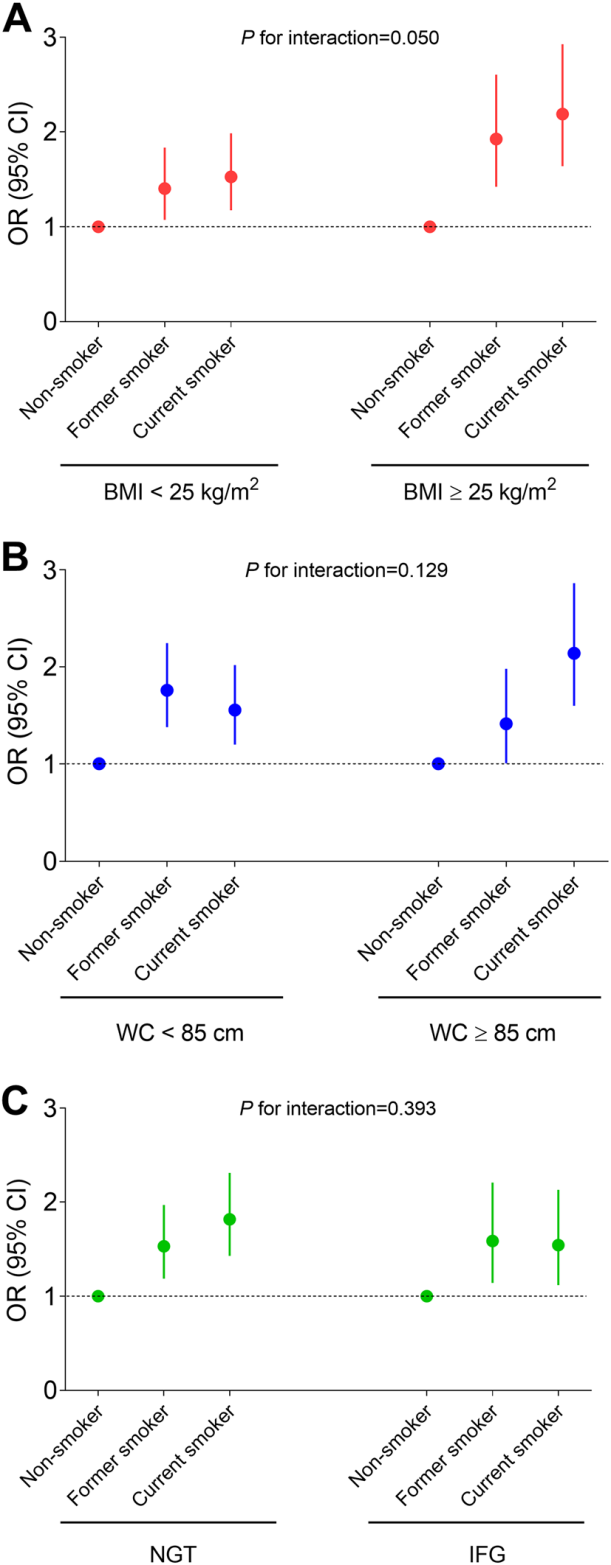
Adjusted odd ratios and 95% confidence intervals for gestational diabetes requiring insulin therapy according to smoking status and prepregnancy body mass index (**A**), waist circumferences (**B**), and baseline glucose tolerance status (**C**). Adjusted for age, alcohol consumption, regular exercise, income status, baseline fasting blood glucose level, family history of diabetes, and dyslipidemia. BMI, body mass index; IFG, impaired fasting glucose; NGT, normal glucose tolerance; WC, waist circumference.

The interaction between smoking and obesity on the risk of insulin-requiring GDM was tested. The effect of smoking did not differ according to general obesity (*P* for interaction = 0.050) and abdominal obesity (*P* for interaction = 0.129). However, the ORs for insulin-requiring GDM were higher in current smokers with general obesity (2.19 [1.64–2.93]) or abdominal obesity (2.14 [1.60–2.86]) compared with other groups (Fig. 2A,B).

The effect of smoking did not differ according to the presence of IFG (*P* for interaction = 0.393; Fig. 2C). Regardless of the presence of pre-pregnancy IFG, former or current smoking was significantly associated with an increased risk of insulin-requiring GDM.

## Discussion

In this study, we found that smoking before pregnancy was associated with an increased risk of GDM requiring insulin therapy. We also found a dose–response relationship between the lifetime amount of smoking and risk of GDM requiring insulin therapy. This association was found consistently in both nonobese and obese women. Although previous studies have reported inconsistent results, our data suggest that prepregnancy smoking should be considered as a significant contributor to insulin-requiring GDM.

Epidemiological studies have tested associations between smoking and risk of DM^15–18^, and some have also investigated the mechanisms underlying these associations^19–21^. Smoking may trigger inflammatory responses, oxidative stress, and insulin resistance^19,20^. For example, an analysis of skeletal muscle biopsy specimens revealed that smokers had decreased expression of peroxisome proliferator-activated receptor-gamma and greater Ser636 phosphorylation of insulin receptor substrate-1 compared with nonsmokers^19,20^. Smoking is also implicated as a risk factor for metabolic syndrome^22^, and metabolic abnormalities may be modulated by the direct negative effect of smoking on insulin resistance. Of the components of metabolic syndrome, high triglyceride and low HDL-cholesterol levels, and abdominal obesity are thought to be the main contributors to this association. A dose-dependent relationship between the number of cigarettes smoked and decreased HDL-cholesterol level and increased triglyceride level has been consistently reported^22^. Although smoking is often linked to reduced body weight, several studies suggest that there are deleterious changes in body composition^23,24^. Other population-based studies have reported a positive association between the amount of smoking and abdominal obesity in current smokers^23^. Greater life-time smoking was significantly associated with higher waist-to-hip ratio and visceral-to-subcutaneous adipose ratio in a population-based cross-sectional study of relatively lean Japanese men^24^. These findings support the general notion that smoking is linked to adverse fat distribution that leads to metabolically unhealthy status. Cigarette smoking also increases exposure to various chemicals and heavy metals such as nicotine, lead, arsenic, and cadmium^19^. This might alter glucose homeostasis and increase the risk of DM^21^.

In a study of US postmenopausal women, those who smoked an average of 16 cigarettes per day had a 1.28-fold higher risk of new diabetes. This risk was mitigated in those who had stopped smoking, and 10 years after smoking cessation, the risk of diabetes became equivalent to that of never-smokers^25^. Our study included young women of childbearing age, and the duration of smoking cessation is probably shorter and, therefore, the risk of GDM in former smokers was similar to or slightly lower than that of current smokers. We found that the total amount of smoking was associated with the risk of GDM requiring insulin therapy, regardless of current or former smoking status.

The prevalence of GDM in Korean women was 5.7% in 2009, 7.8% in 2010, and 9.5% in 2011^26^. About 5% of women with GDM took medication during pregnancy, and more than 98% of the Korean patients with GDM administered insulin^26^. In our study, women with GDM being treated with insulin accounted for 0.6% of all pregnant women, which is similar to the statistics for GDM in Korea. The debate about GDM screening and diagnostic methods persists in global academic circles and professional societies. It is important to identify factors related to the development of severe GDM and pay more attention to them. In our study, we used the administration of insulin to control blood glucose level as an indicator of severe GDM, and we found that the amount of smoking was an independent risk factor for severe GDM.

The key strengths of this study include the enrollment of the large study population of > 300,000 deliveries. Because of national health insurance coverage, almost all pregnant women in Korea undergo GDM screening and treatment during pregnancy. Therefore, our study included almost all women who underwent a health examination within 1 year of conception. The dose–response relationship between smoking and GDM was explained using detailed smoking indices. A dose–response relationship is usually considered to be an evidence in support of causality. However, we acknowledge some limitations. Because smoking status was obtained from self-administered questionnaires, we cannot exclude the possibility that misreporting led to some individuals being misclassified with regard to their smoking status. Considering the large number of participants, we believe that misclassification had only very limited influence on the results obtained; earlier studies have also reported low misclassification rates of smoking status^11,27^. Second, our reported relationship between smoking and GDM requiring insulin therapy was likely to have been attenuated by the inclusion of women with mild GDM who were treated with diet and exercise in the control group. The higher risk of severe GDM among pregnant women who smoke could be mediated through the same pathophysiologic mechanisms that underlie the higher risk of diabetes in people who smoke, which includes insulin resistance and impaired glucose homeostasis. Therefore, we can assume that the same correlation may be seen in women with mild GDM who were treated with diet and exercise. Recently, it has been reported that prenatal smoking is associated with a higher risk of GDM, independent of the treatment modality of GDM^28^. Third, we had no data on changes in smoking status during pregnancy. The prevalence of active smoking among pregnant women may be even lower because women smokers may stop during pregnancy. In our study, increasing the amount of smoking, regardless of being a former or current smoker before pregnancy, influenced the risk of GDM requiring insulin therapy. Lastly, data on passive smoking were not available in the NHIS database. Exposure to secondhand smoke has been reported to be associated with a higher risk of several types of cancer and cardiovascular disease^29^. It would be interesting to examine the effect of passive smoking on the risk of GDM.

In conclusion, in a cohort of 325,297 women in Korea, former and current smokers had a significantly increased risk for GDM requiring insulin therapy and the risk increased with the number of cigarettes smoked, regardless of the current smoking status. The risk of insulin-requiring GDM was 2.3 times higher in women who smoked > 10 pack-years than in nonsmokers. Of note, even ≤ 2 pack-years of smoking increased the risk by 50%. Cessation of smoking should be emphasized in women of childbearing age because cumulative lifetime smoking is a major contributing factor to insulin-requiring GDM.
